# Study on regeneration of waste powder activated carbon through pyrolysis and its adsorption capacity of phosphorus

**DOI:** 10.1038/s41598-017-19131-x

**Published:** 2018-01-15

**Authors:** Yi Li, Hailan Jin, Wenbo Liu, Hang Su, Yao Lu, Jianfen Li

**Affiliations:** 1 College of Material Science and Engineering, Northeast Forestry University, Harbin, 150040 PR China; 2 School of Chemical and Environmental Engineering, Wuhan Polytechnic University, Wuhan, 430073 PR China

## Abstract

The regeneration of WPAC through pyrolysis and its adsorption capacity of phosphorus were studied. The optimum conditions for WPAC regeneration were 650 °C and 2 h which resulted in a recovery of BET surface and total pore volume with a value of 1161.4 m^2^/g and 1.2176 m^3^/g. WPAC had a maximum PO_4_^3−^-P adsorption capacity of 9.65 mg/g which was 48.93% of PAC, while RWPAC had a maximum PO_4_^3−^-P adsorption capacity of 15.31 mg/g which was 77.64% of PAC. The kinetic analysis revealed that Langmuir model could well describe the adsorption process of PAC, WPAC and RWPAC on PO_4_^3−^-P and the PO_4_^3−^-P adsorption followed the pseudo-second-order model.

## Introduction

Phosphorous is the main component causing the eutrophication in lakes, coastal areas, and other confined water bodies. Many technologies including adsorption, biological nutrient removal and precipitation have been applied to remove phosphorus from wastewater due to the increasingly stringent regulations on phosphorus discharge. The adsorption process is one of the most efficient methods of removing phosphorus from wastewater.

Powder activated carbons (PACs) is the most widelyused adsorbent due to its large surface area, well developed pore structure and adsorption capacity^1,2^. However, during their applications, PACs will become progressively saturated with adsorbates, and therefore lose their adsorption capacity. Usually those waste PACs (WPAC) are incinerated or discharged which will cause a secondary source of pollution^3^. Compared with incineration or other treatments, regeneration of WPAC which can restore their adsorption capacity is more significant for the waste re-utilization and environmental protection.

There are several kinds of methods for activated carbon regeneration like oxidizing regeneration^4^, thermal regeneration^5,6^, microwave regeneration^7,8^ and biological regeneration^9,10^. However, biological regeneration and oxidizing method are either time-consuming or uneconomic. Although microwave has been utilized to regenerate activated carbon while the application is still very limited. The most widely used regeneration method is thermal regeneration. Three PACs with widely different BET areas were evaluated to determine the effects of thermal regeneration on their physical and chemical properties by Clifford^11^. PAC weight% recoveries were in the range of 60–80%, and the recovery of wastewater adsorption capacity regularly exceeded 100% for all carbons. The pyrolysis and combustion characteristics of a waste oil absorbing activated carbon were investigated using a thermogravimetric analyzer under heating rate of 10 °C/min from 25 °C to 1000 °C. The results indicated that the pyrolytic process is divided into three stages while the incineration process of the waste oil absorbing activated carbon is in four phases^12^. The regeneration process of WPAC is depended on a large number of parameters like regeneration duration and the temperature which will make great influence on adsorption capacity recovery percentage and weight loss percentage. Therefore, the analysis of the regeneration process and the evaluation of regeneration effect are strongly required.

Pyrolysis is regarded to be a promising approach in which organic matters can be decomposed and converted into biochar, bio-oil and permanent gases in the absence of oxygen during 150~700 °C^13^. The focus of the present work is to regenerate the WPAC by pyrolysis. The effects of regeneration on the characteristic of WPAC through pyrolysis were analyzed. Meanwhile the performance for the phosphorous adsorption by WRPAC was also investigated.

## Materials and Method

### Materials

The fresh PAC and WPAC used for the experiment were obtained from a saccharin plant in XiangYang, HuBei, China. PAC was used as the adsorbent to deal with the saccharin wastewater with a mean value of COD (18.2 g/L), NH_4_^+^-N (50 mg/L) and pH(3.6). PAC was previously boiled with deionized water for 1.5 h in order to remove the other impurities. Then PAC and WPAC were dried to constant weight for the subsequent use.

### Regeneration procedures

The regeneration of WPAC was carried out in a vertical pyrolysis furnace. Experiments were conducted using batch method to determine the optimal temperature and regeneration time. To determine the optimal temperature of pyrolysis, about 10 g of the sample was pyrolysed under 500 mL/min N_2_ flow at heating rate of 10 °C/min from 25 °C to the designated temperature (550 °C, 600 °C, 650 °C, 700 °C and 750 °C) with a regeneration durationranged from 0.5 to 3.0 h. The atmosphere was also maintained during the heating up and cooling-down intervals. The loss rate of pyrolysis *η*(LRP) was calculated according to the Eq. (1).

1 $${\eta }=\frac{{M}_{1}-{M}_{2}}{{M}_{1}}\times 100 \% $$

where ${M}_{1}$ was the mass of WPAC before regeneration, ${M}_{2}$ was the mass of WPAC after regeneration.

### Phosphorus adsorption capacity of WPAC

Experiments were carried out to investigate the adsorption capacity of PAC, WPAC and RWPAC on phosphorus.

#### Adsorption isotherms

Artificial wastewater was synthesized by adding a certain amount of KH_2_PO_4_ into distilled water. Equilibrium sorption studies were conducted in a 50 mL erlenmeyer flasks each containing 30 mL wastewater. 0.02 g PAC, WPAC or RWPAC with a corresponding initial concentration of wastewater (10, 40, 80, 120, 200 and 400 mgPO_4_^3−^-P/L^−1^, respectively). The flasks were agitated in a shaker at 150 rpm and 20 °C for 4 h to reach equilibrium. The PO_4_^3−^-P adsorption capacity per unit mass of adsorbent at equilibrium, ${q}_{e}$(mgPO_4_^3−^-P/g), was calculated by Eq. (2):

2 $${q}_{e}=({C}_{0}-C)V/(1000\,M)$$

where V was the volume of the solution (mL); M was the mass of adsorbent (g); ${C}_{0}$ and $C$ were the concentration of PO_4_^3−^-P before and after the adsorption(mg /L).

#### Adsorption isotherms model

Adsorption isotherms is necessary to describe the adsorption capacity of PAC, WPAC and RWPAC. The equilibrium concentration of PAC, WPAC and RWPAC in the solution could be helpful for the analysis and the design of the sorption systems. In this study, two adsorption isotherms were developed by Langmuir model (Eq. (3)) and Freundlich model (Eq. (4))^14,15^.

3 $$\frac{{C}_{e}}{{q}_{e}}=\frac{1}{{q}_{{\rm{\max }}}{k}_{L}}+\frac{{C}_{e}}{{q}_{{\rm{\max }}}}$$

4 $$\mathrm{log}\,{q}_{e}=\,\mathrm{log}\,{k}_{F}+\frac{1}{n}\,\mathrm{log}\,{C}_{e}$$

where ${k}_{L}$ is the Langmuir isotherm constant (L/mg), ${q}_{\max }$ represent the maximum PO_4_^3−^-P adsorption capacity of PAC, WPAC and RWPAC (mg/g). ${k}_{F}$ is the Freundlich isotherm constant which indicates the maximum adsorption capacity (mg/g). 1/n is the Freundlich isotherm constant which is dimensionless. ${C}_{e}$ is the equilibrium concentration of the adsorbed substance in the liquid phase (mg/L) and ${q}_{e}$ is the constant that indicates the maximum adsorbate quantity of the adsorbent (mg/g).

### Analytical methods

TG and derivative thermogravimetric (DTG) analysis was carried out with a thermogravimetric analyzer (TG/DTA SDT Q600, USA). The chemical functional groups in the samples were investigated by FTIR (Nicolet 6700, Thermo Fisher Scientific, USA). The surface area and pore characteristics of WPAC were analyzed using BET(ASAP2020 HD88, USA). Phosphate concentration was determined by the colorimetric method. All of the tests were conducted in 3 replications, and the mean values were used.

## Results and Discussion

### Influence of regeneration conditions

**Figure 1:**
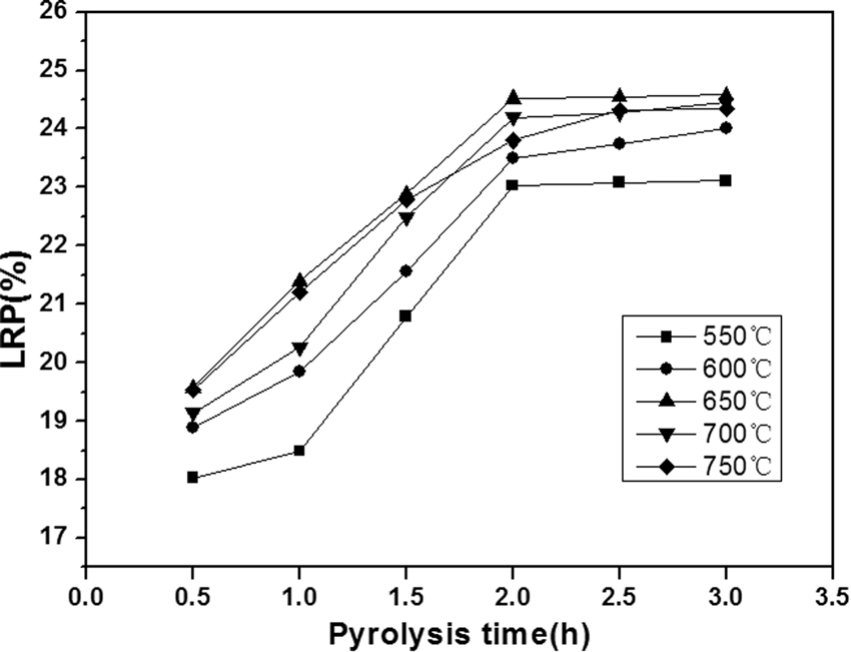
Influence of regeneration conditions on the LRP.

The regeneration temperature and duration of pyrolysis are important parameters for WPAC regeneration, as organic matters can be decomposed at high temperature. Besides, the regeneration temperature and duration also make influence on the weight loss percentage. The influences of pyrolysis duration and pyrolysis temperature on the LRP of WPAC were displayed on Fig. 1. As seen in Fig. 1, LRP increased with the extend of pyrolysis time for all pyrolysis temperatures. The maximum LRP was obtained at 2 h. After 2 h, there was not obvious difference on LRP. For the same pyrolysis time, LRP increased at the region of 550–650 °C while litter difference was caused at the region of 650–750 °C. The maximum LRP (24.5%) was reached at 650 °C which indicated that the decomposition of organic matters adsorbed on the WPAC was completely finished. Therefore, the optimum regeneration conditions were 650 °C and 2 h with the concern of energy consumption and economic for regeneration.

### Characteristic of WPAC under different regeneration conditions

**Figure 2:**
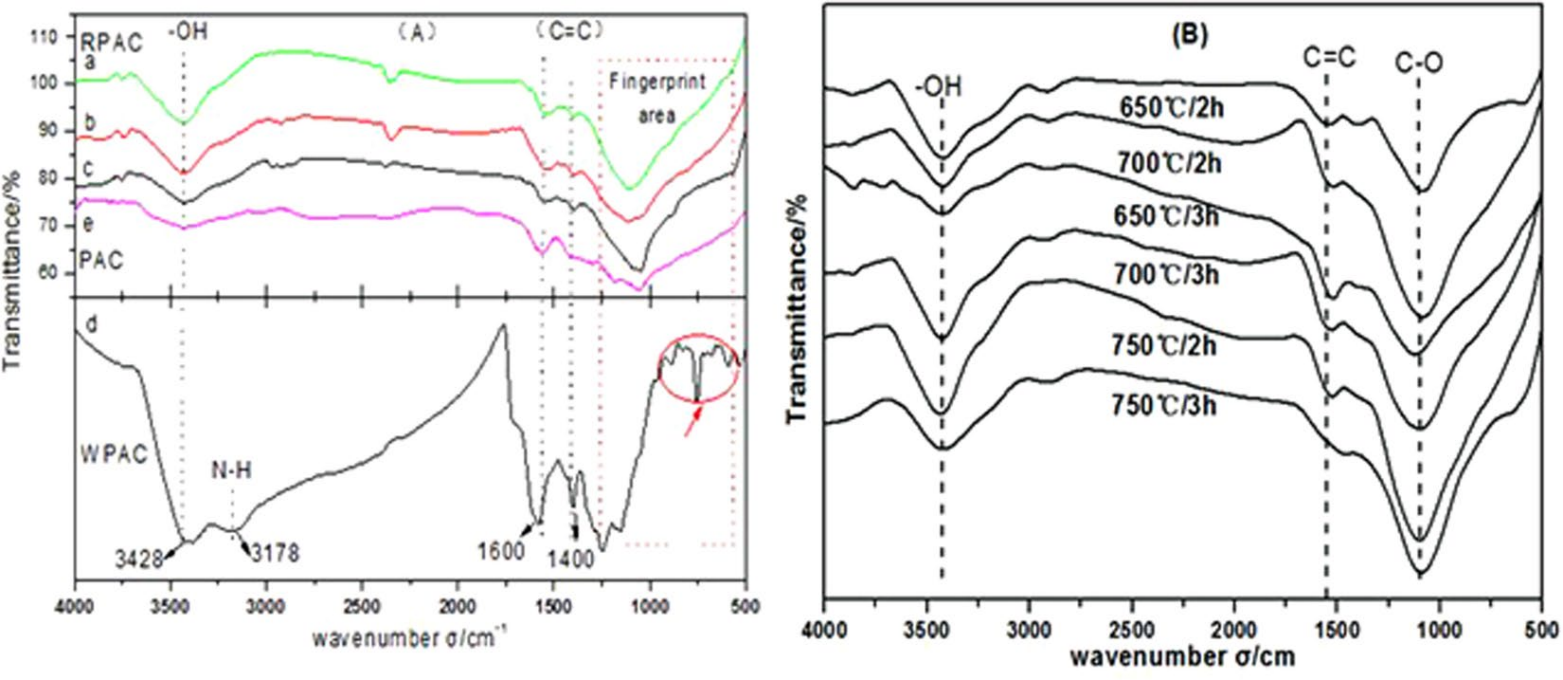
Characteristic of WPAC (**A**) FTIR of WPAC before and after regeneration; (**B**) FTIR of WPAC under different regeneration conditions. a: 650 °C, 2 h; b: 650 °C, 3 h; c: 700 °C, 2 h, d: WPAC; e: PAC.

Changes in surface functional groups are also reflected by the FTIR spectra. The FTIR spectra of WPAC before and after regeneration are presented in Fig. 2. The relevant peaks attributed to special functional groups and compounds are summarized in Table 1. The peaks at wave numbers of 3428, 3178, 1400–1600, 500–1000 cm^−1^ showed the surface carbon structure of the WPAC. This structure consists of chain hydrocarbons and functional groups such as anthranilic acid, methyl anthranilate and methyl ortho-chlorobenzoate. Peaks at 3178 cm^−1^ and 500–1000 cm^−1^ were disappeared. Peaks at 3428 cm^−1^, 1600–1400 cm^−1^ and 1000–1300 cm^−1^ were weaken. It indicated that the anthranilic acid and anthranilic acid methyl ester adsorbed on the WPAC had been effectively decomposed after pyrolysis regeneration. Meanwhile, the spectra was very sensitive to the changes of molecular structure in the fingerprint area. The peaks disappeared in the fingerprint area indicated that the organic matters adsorbed by the PAC had been decomposed. It also could be seen that the organic matters adsorbed on the WPAC had been effectively decomposed at 650 °C after 2 h regeneration.

**Table 1:** Analysis of the peaks of FTIR spectra.

Peak position/cm^−1^	Functional group	Before regeneration	After regeneration
3428 cm^−1^	−OH	Strong	Weaken
3178 cm^−1^	−NH−/−NH_2_−	Strong	Disappear
1600–1400 cm^−1^	C=C	Strong	Weaken
1000–1300 cm^−1^	C-O	Strong	Weaken
500–1000 cm^−1^ (fingerprint)	—	Complicated	Disappear

**Figure 3:**
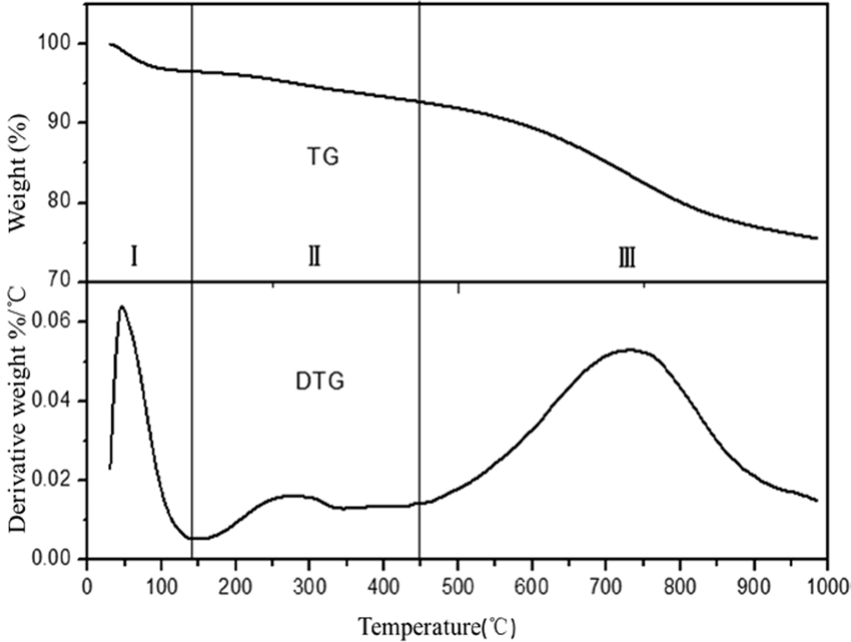
TG/DTG curve of RWPAC.

Figure 3 showed the TG and DTG pyrolysis profiles of WPAC. The TG-DTG curves showed that mass of the sample was lost gradually and the decomposition process could be divided into three phases: moisture desorption, thermal desorption of organic and inorganic matters adsorbed by activated carbon, and finally carbonization of solid pyrolysis residue.

1. The first weight loss step is from 50~110 °C which was due to the water emission. It is well known that some of the water in the sample and the boiling point of the water is relatively low which could be easily stripped under low temperature.
2. The second weight loss step is from 200 °C to 350 °C, this step was considered as the dehydration of oxygen containing functional groups.
3. The third weight loss step is from 600~800 °C which was the main pyrolysis stage for the pyrolysis of organic compounds adsorbed on the WPAC.

Table 2 showed the BET surface and pore volume of PAC, WPAC and RWPAC. It could be seen that there were obvious differences between WPAC and RWPAC. PAC had a large BET surface which could be reached to 1228.6 m^2^/g. While WPAC only had 593.2 m^2^/g which was 48.3% of PAC. After 2 h regeneration at 650 °C, BET surface of RWPAC had been recovered to 1161.4 m^2^/g which was 94.5% of PAC. Compared with the total pore volume of PAC (1.2380 m^3^/g), the total pore volume of WPAC was 0.4932 m^3^/g while RWPAC had a total pore volume of 1.2176 m^3^/g. RWPAC can keep its porous structure and has a relative high specific surface area after regenerated by pyrolysis method. These results indicated pyrolysis was an effective way to recover the WPAC and the characteristic of WPAC could be recovered which was benefit for re-utilization of WPAC.

**Table 2:** BET surface and pore volume of PAC,WPAC and RPAC.

sample	PAC	WPAC	RWPAC
BET surface area (m^2^·g^−1^)	1228.6	593.2	1161.4
total pore volume (m^3^/g)	1.2380	0.4932	1.2176
t-Plot micropore volume (m^3^/g)	0.2545	0.0991	0.2232
Mesoporous volumes (m^3^/g)	0.8737	0.3015	0.8026

### Phosphorus adsorption

**Figure 4:**
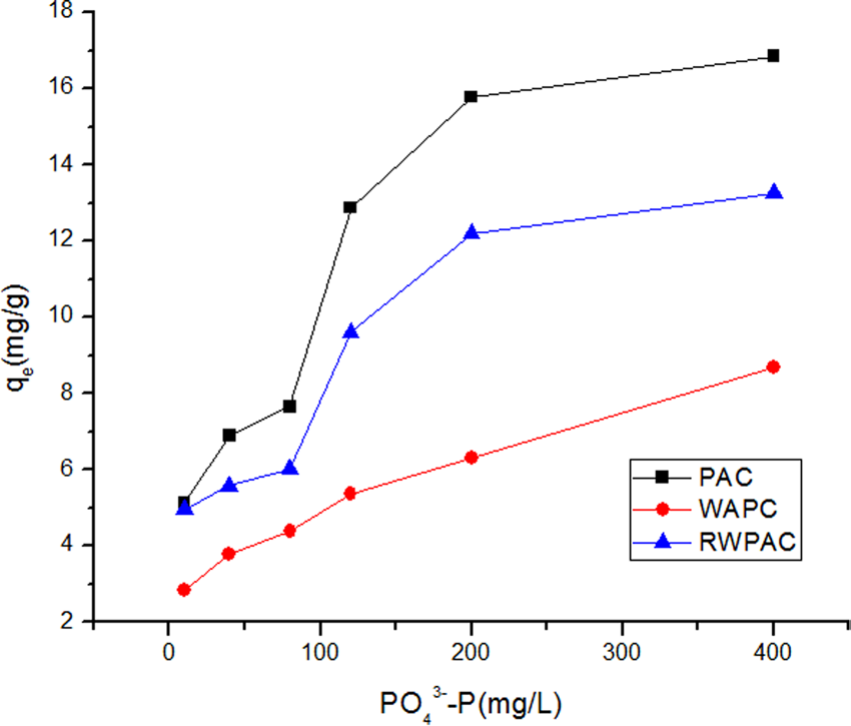
Adsorption performance of PAC, WPAC and RWPAC on phosphorus (P).

For evaluation of regenerated WPAC, the adsorption performance of PO_4_^3−^-P was investigated. In this study, the adsorption capacity of PAC, WPAC and RWPAC was tested by varying the initial concentration of phosphorus (P) (20 °C). Figure 4 show the general trend is such that the adsorption capacity increases with the increase in the initial P concentration until reaching the saturated state. The maximum adsorption capacity of PAC, WPAC and RWPAC were 16.86 mgPO_4_^3−^-P/g, 8.70 mgPO_4_^3−^-P/g. and 13.27 mgPO_4_^3−^-P/g respectively.

### Adsorption isotherms and kinetic modeling

#### Adsorption isotherms

In order to evaluate the difference in regeneration on enhancing the adsorption performance of WPAC, it was necessary to develop a similar equilibrium to provide a better comparison and understanding of the adsorption process. For this study, the isotherm parameters of two models calculated from the slope and the intercept of the plots were given in Table 3. The results indicated that the adsorption of PAC, WPAC and RWPAC on P could be well described by Langmuir model (R^2^ > 0.943) compared with the Freundlich model (R^2^ > 0.83). The Langmuir isotherm model is based on the assumption that all adsorption sites are alike and equally energetic, making the surface homogeneous^16^. The PO_4_^3−^-P adsorption mechanism by PAC was single-layer adsorption. From the fitting of experimental data using the Langmuir isotherm, it was found that there were consistent between the model-fitted and experimentally measured adsorption capacities. According to the calculation results of Langmuir model, PAC used in this study had a maximum P adsorption capacity of 19.72 mg/g. It is higher than the results of Li who used the powder activated carbon(PAC) prepared from rice husks as the adsorbent of phosphorus(P) which had a saturated adsorption capacity of 6.93 mg/g^17^. The lower specific surface area(BET) of powder activated carbon (886.3 m^2^/g) resulted in a lower adsorption capacity compared with RWPAC (1161.4 m^2^/g) in this paper. WPAC had a maximum PO_4_^3−^-P adsorption capacity of 9.65 mg/g which was 48.93% of fresh PAC. While RWPAC had a maximum PO_4_^3−^-P adsorption capacity of 15.31 mg/g which was 77.64% of PAC. It indicated that the adsorption capacity had been greatly recovered after regeneration.

**Table 3:** Isotherms parameters of Langmuir and Freundlich models for PAC, WPAC and RWPAC.

Object	Langmuir parameter			Freundlich parameter		
	q_max_(mg/g)	k_L_(L/mg)	R^2^	k_F_(mg/g)	1/n	R^2^
PAC	19.72	0.014	0.9504	2.03	0.1188	0.8993
WPAC	9.65	0.014	0.9496	1.99	0.3586	0.8609
RWPAC	15.31	0.015	0.9479	2.13	0.2998	0.8337

#### Adsorption kinetics

To investigate the mechanism involved in the P adsorption onto the PAC, WPAC and RWPAC, the pseudo-second-order kinetic model and the intraparticle model were used for the analysis of P adsorption.

The pseudo-second-order equation is expressed as^18^:

5 $$\frac{t}{{q}_{t}}=\frac{1}{{k}_{1}{{q}_{e}}^{2}}+\frac{1}{{q}_{e}}t$$

The intraparticle diffusion equation is expressed as^19^:

6 $${q}_{t}={k}_{2}{t}^{1/2}+d$$

where, ${k}_{1}$ and ${k}_{2}$ represented the rate constant of the pseudo-second-order model (g/min mg) and the intraparticle diffusion rate constant (mg/min^1/2^ g), respectively. The ${q}_{e}$ and ${q}_{t}$ (mg/g) are the quantity of PO_4_^3−^-P adsorbed at equilibrium and at time t, respectively, with d as the intercept.

Based on Eqs (5) and (6), the values of k_1_ and q_e_ could be obtained from the straight-line plot for t/qt against t (Table 4). The kinetics of PAC, WPAC and RWPAC on phosphorus (P) were very fast which could be reached to pseudo-equilibrium in the 3 h. These results confirmed that PAC was an attractive adsorbent which was widely used in wastewater treatments because of its large surface area and well developed pore.

**Table 4:** Kinetic matters for P adsorption using pseudo-second-order kinetic model and the intra-particle model.

	pseudo-second-order kinetic model			intra-particle model	
	k_1_ (g/min mg)	q_e_ (mg/g)	R^2^	k_2_ (mg/min^1/2^ g)	R^2^
PAC	0.48	19.05	0.9484	1.0731	0.8934
WPAC	1.6	7.10	0.9870	0.4416	0.9425
RWPAC	0.78	13.46	0.9618	0.7981	0.8826

**Figure 5:**
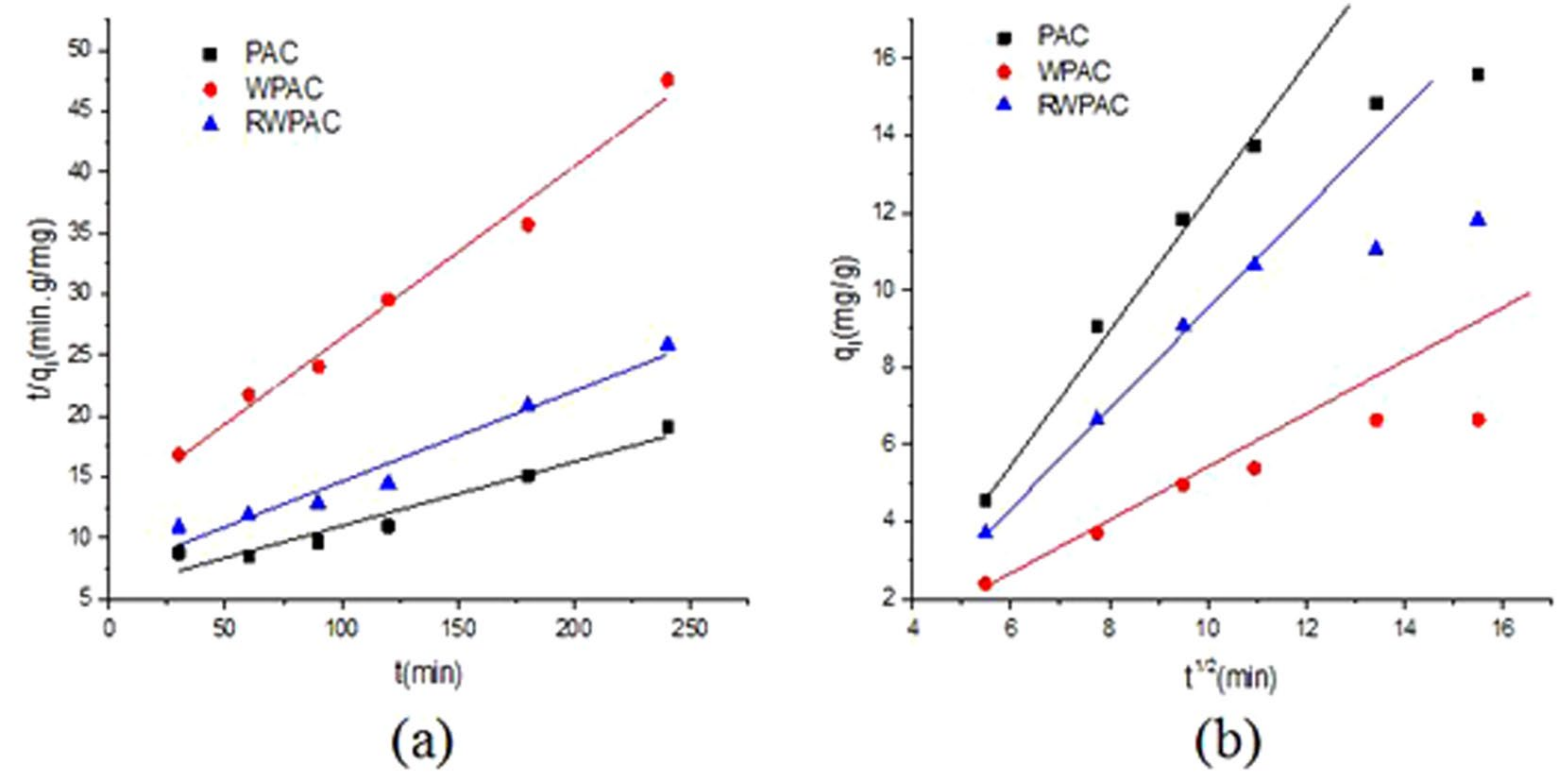
(**a**) Pseudo-second-order kinetic models for P adsorption by PAC, WPAC and RWPAC. (**b**) Intraparticle diffusion models for P adsorption by PAC, WPAC and RWPAC.

The plots for the pseudo-second-order kinetic and the intraparticle diffusion model fitted with the experimental data for PAC, WPAC and RWPAC were shown in Fig. 5(a) and (b). Comparing the correlation coefficients listed in Table 3, it was found that the adsorption of PO_4_^3−^-P by PAC, WPAC and RWPAC could be well described by the pseudo-second-order kinetic model, which has a higher correlation coefficient value (R^2^ > 0.94). The Padsorbed on the PAC, WPAC and RWPAC involves two stages (surface sorption and intraparticle diffusion). The intraparticle diffusion model can only be applied to describe the first linear stage of PO_4_^3−^-P, which is probably due to the boundary-layer diffusion effect, whereas the final linear portion may be due to the intraparticle effect^20^.

### Benefit of the regeneration by pyrolysis

The WPAC could be regenerated through pyrolysis which had several advantages: first, it cut down the consumption of the coal and the natural resources; secondly, it reduced the secondary pollution by the WPAC; finally, it greatly cut down the cost of wastewater treatment. The energy demands of regeneration was mainly consumed by the pyrolysis furnace. The consumption of electric by pyrolysis furnace was about 40 kwh to regenerate 1 ton WPAC, while the consumption of electric to produce 1 ton PAC was about 160 kwh. It could be seen that the energy demands of regeneration had been greatly decreased which was about 25% of the energy demands required to produce new PAC.

## Conclusion

Through this study, it was found that waste power activated carbon could be recovered by pyrolysis regeneration method.The optimum regeneration conditions were 650 °C and 2 h. Under this condition, BET surface of RWPAC had been recovered to 1161.4 m^2^/g which was 94.5% of PAC. The pyrolysis of WPAC consists three stages: moisture emission, dehydration of oxygen containing functional groups and carbonization. The performance of PO_4_^3−^-P adsorption were also studied to evaluate the regeneration process. The PO_4_^3−^-P adsorption capacity recovery percentage could increased from 48.93% (WPAC) to 77.64% (RWPAC) of fresh PAC. This regeneration method can recover the characteristic of WPAC to ensure the adsorption capacity and decrease the risk of secondary pollution in the activated carbon regeneration process.
